# Successful development of molecular diagnostic technology combining mini-barcoding and high-resolution melting for traditional Chinese medicine agarwood species based on single-nucleotide polymorphism in the chloroplast genome

**DOI:** 10.3389/fpls.2024.1405168

**Published:** 2024-07-31

**Authors:** Jian Feng, Yangyang Liu, Anzhen Xie, Yun Yang, Feifei Lv, Jianhe Wei

**Affiliations:** ^1^ Hainan Provincial Key Laboratory of Resources Conservation and Development of Southern Medicine & International Joint Research Center for Quality of Traditional Chinese Medicine & Key Laboratory of State Administration of Traditional Chinese Medicine for Agarwood Sustainable Utilization, Hainan Branch of the Institute of Medicinal Plant Development, Chinese Academy of Medical Sciences and Peking Union Medical College, Haikou, China; ^2^ Key Laboratory of Bioactive Substances and Resources Utilization of Chinese Herbal Medicine, Ministry of Education & National Engineering Laboratory for Breeding of Endangered Medicinal Materials, Institute of Medicinal Plant Development, Chinese Academy of Medical Sciences and Peking Union Medical College, Beijing, China

**Keywords:** agarwood, traditional Chinese medicine, mini-barcoding, high-resolution melting, SNP

## Abstract

Agarwood is a valuable traditional medicine and fragrance. The production process is a typical injury-induced defense response. Currently, there are approximately 22 known species in the genus *Aquilaria* Lam., all of which can produce agarwood, whereas there are only two legal species of traditional Chinese medicinal agarwood, *Aquilaria sinensis* (Lour.) Spreng. and *Aquilaria agallocha* (Lour.) Roxb. The Taiwan herbal Pharmacopoeia of China stipulates that the medicinal agarwood species are *A. sinensis* and its relatives in the same genus. Moreover, there are five species of agarwood available for clinical medicinal use in Japan, including *A. agallocha* and *A. sinensis*, which are often confused with each other or used in a mixed way in the trade process. Therefore, accurate identification of traditional Chinese medicinal agarwood species is important to ensure the authenticity of traditional medicines and to guide the safety of clinical medication. In this study, 59 specific single-nucleotide polymorphism loci were screened and obtained from the chloroplast genomes of 12 species of the genus *Aquilaria* Lam. We established an identification method for traditional Chinese medicinal agarwood using mini-barcoding combined with high-resolution melting (HRM) and designed and validated 10 pairs of primers from the *psb*M-*trn*D, *psb*A, *rps*16, *pet*N, *ndh*E-*psa*C, *rps*4, *atp*E, *ycf*1, *rps*15-*trn*N, and *mat*K regions. The amplification products were all less than 200 bp, with a high success rate of amplification. The method was applied to successfully identify traditional Chinese medicinal agarwood species from commercial agarwood samples. Overall, the sensitivity of this method was sufficient to detect 1% of adulterants in medicinal agarwood products, proving that mini-barcoding HRM is a powerful and flexible tool. This method can be used as a fast and effective high-throughput method for authenticity testing of traditional Chinese medicinal agarwood and its raw materials containing agarwood-containing proprietary Chinese medicines and is recommended for industrial applications.

## 1 Introduction

Agarwood is the resinous wood of the species of genus *Aquilaria* Lam. of the family Thymalaeaceae. The natural distribution area of agarwood includes China (Hainan Island, Yunnan, Guangdong, and Fujian province), Laos, Cambodia, Vietnam, Indonesia, and Malaysia (Wyn and Anak, 2010; Tan et al., 2019). Twenty-two species of agarwood have been recorded worldwide (Michael, 2023). The use of agarwood in trade markets has substantially increased, and, consequently, its supply is becoming increasingly exhausted. All species of *Aquilaria* Lam. genus have been listed in Appendix II of the Convention on International Trade in Endangered Species of Wild Fauna and Flora (CITES) since 2005 (Lee and Mohamed, 2016; The Endangered Species Import and Export Management Office of the P. R. China, 2023), as well as in the Red Book of Endangered Plants of the World Conservation Union.

Agarwood has a long history of use as a traditional medicine and fragrance (Ye et al., 2016; Liu et al., 2017). Indeed, in ancient Egypt, agarwood was used for embalming corpses; in ancient India, it was used for hair fumigation; and in the Middle East, it was used in large quantities for burning (Lee and Mohamed, 2016; Liu, 2020). Agarwood is also the main component of Japanese “Kyushin” medicine. The earliest medical use of agarwood can be traced back to *Míng Yī Bié Lù*, which was written by the táo of the Liang Dynasty in China (Tao et al., 1986). Only a few species of agarwood have been documented for use as clinical medicines; two species of *Aquilaria sinensis* (Lour.) Spreng. and *Aquilaria agallocha* (Lour.) Roxb. are available for clinical use in China (State Food and Drug Administration, 2004; Chinese Pharmacopoeia Commission, 2020). In addition, the Taiwan Herbal Pharmacopoeia of China stipulates that the medicinal agarwood species are *A. sinensis* and its relatives in the same genus (Chen et al., 2021). In addition, five species of agarwood (*A. agallocha*, *A. crassna* Pierre, *A. malaccensis* Lam., *A. sinensis*, and *A. filaria* Oken Merr.) are available for clinical use in Japan (Pharmaceutical review and control division, 2022). The classification of the genus *Aquilaria* Lam. has been of great interest to taxonomists. Most plants in the genus *Aquilaria* Lam. have multiple synonyms; for example, the synonyms of medicinal *A. sinensis* are *A. sinensis* (Lour.) Gilg, *A. sinensis* (Lour.) Spreng., *A. sinensis* (Lour.) Merr., and *A. chinensis* Spreng. The synonyms of *A. agallocha* are *A. agallocha* Roxb., *A. agallocha* (Lour.) Roxb. Ex Finl., *Agallocha malaccensis* (Lam.) Kuntze, and *Aloexylum agallocha* Lour (López-Sampson and Page, 2018; GBIF Secretariat, 2023; Michael, 2023). Although they have multiple synonyms, their genetic information is the same, and all of them can be used as the genetic information of the same species.

Verifying that the species of Chinese herbal medicines used in Chinese medicine clinics are reported accurately is the key to ensuring the safety of the medicines used (Shen et al., 2022; Chen et al., 2023). Some traders, in order to profit from the trading market, adulterate agarwood or substitutes because agarwood differs from the appearance of the traits cannot be distinguished from the species. Therefore, it is of great importance to identify medicinal agarwood species from large amounts of agarwood. Some scholars have suggested *ITS*2+*trn*L-*trn*F (Lee et al., 2016), *ITS*2+*mat*K (Kang et al., 2019), *ITS*+*mat*K, ITS+*rbc*L, *ITS*+*trn*L-*trn*F (Li et al., 2019; Lin et al., 2023), and *psb*J-*pet*A+*trn*T-*trn*L (Hishamuddin et al., 2023) for the molecular identification of agarwood. However, the actual situation is not working well. Under various conditions (such as induction or storage), the DNA of the agarwood was highly degraded and was not easily amplified, and it was difficult to identify it using a barcode combination such as plant DNA. In addition, when DNA barcodes were used for identification of agarwood, it took a long time to extract, amplify, sequence, and analyze the samples. Mohamed et al. (2012) and Lee et al. (2016) used RT-PCR to identify DNA-degraded agarwood samples, but the method still had low sensitivity and specificity. Therefore, the difference in mini-barcoding and melting curves represents a new method for the identification of low-quality DNA or highly degradable DNA in medicinal materials, and a rapid and efficient identification method is urgently required.

DNA mini-barcoding is the development of shorter molecular markers (usually 100–300 bp) based on the nucleotide profile of a specific species or genus group, effectively solving the problem of identifying herbs with varying degrees of DNA degradation (Yeo et al., 2020; Parveen et al., 2022). High-resolution melting (HRM) is a classical melting curve analysis method based on PCR fragments, which, compared to other molecular identification methods, allows the sample to be identified, even if a single-base variation occurs in the DNA sequence, facilitating the quantitative determination of the rate of adulteration (Wu et al., 2008; Ganopoulos et al., 2011; Duan et al., 2018; Buddhachat et al., 2021). HRM represents a simple, sensitive, low cost, and specific method that can realize closed-tube operation (Reed et al., 2007; Kalivas et al., 2014; Kim et al., 2023). Lee et al. (2019) used barcode-HRM to validate the origin and adulterant of agarwood species, but there is still a need for accurate identification of agarwood species, especially for proprietary medicines.

The development of high-throughput sequencing technology has allowed the chloroplast and chromosome-level genomes of more species to be sequenced. Unique single-nucleotide polymorphism (SNP) loci of species can be obtained from these data, allowing the precise identification of species (Kim et al., 2022; Fei et al., 2023). However, the identification of agarwood species plays a crucial role in clinical practice, forestry, the daily chemical industry, and the CITES. Therefore, in this study, mini-barcoding combined with HRM was identified as a successful molecular diagnostic technology for identifying traditional Chinese medicinal agarwood species based on chloroplast genomes.

## 2 Materials and methods

### 2.1 Sample collection and preparation

Twenty four batches of agarwood and its products were included in this study (**Table 1**). Four batches of dry leaves of *A. sinensis*, *A. agallocha*, *A. crassna*, and *A. subintegra* were collected from the South medicinal botanical garden. Twenty batches of commercially available agarwood products (such as wood patch, chips, blocks, powder, and Chinese patent medicines) were collected. All materials were identified by Professor Yangyang Liu, and all specimens were kept in the agarwood identification center herbarium.

**Table 1:** The agarwood and products used in this study.

Name	Type	Trade species	Sampling location	Mini-barcoding HRM species identification results (medicinal agrwood or not)
AS01	Dry leaf	*A. sinensis*	South medicinal botanical garden, Haikou, China	Medicinal agrwood/*A. sinensis*
AM01	Dry leaf	*A. agallocha*		Medicinal agrwood/*A. agallocha*
AC01	Dry leaf	*A. crassna*		Non-medicinal agarwood
ASU01	Dry leaf	*A. subintegra*		Non-medicinal agarwood
C001	Wood chips	*A. sinensis*	Hainan, China	Medicinal agrwood/*A. sinensis*
C002	Wood chips	*A. sinensis*	Huazhou, China	Medicinal agrwood/*A. sinensis*
C003	Wood powder (supercritical residue)	unknown	Dianbai, China	Medicinal agrwood/*A. sinensis*
C004	Wood powder (hot reflux residue)	unknown	Haikou, China	Medicinal agrwood/*A. sinensis*
C005	Wood patch	unknown	Bozhou, China	Non-medicinal agarwood
C006	Wood patch	unknown	Vietnam	Non-medicinal agarwood
C007	Wood patch	unknown	Malaysia	Non-medicinal agarwood
C008	Wood patch	unknown	Vietnam	Non-medicinal agarwood
C009	Wood patch	unknown	Dongguan, China	Non-medicinal agarwood
C010	Wood patch	unknown	Dongguan, China	Non-medicinal agarwood
C011	Wood patch	unknown	Yunnan, China	Medicinal agrwood/*A. sinensis*
C012	Wood patch	unknown	Thailand	Non-medicinal agarwood
C013	Wood patch	unknown	Malaysia	Non-medicinal agarwood
C014	Wood chips	unknown	Bangladesh	Medicinal agrwood/*A. agallocha*
C015	Wood block	unknown	Kalimantan	Non-medicinal agarwood
C016	Wood block	unknown	Papua New Guinea	Medicinal agrwood/*A. sinensis*
C017	Chinese patent medicine A	unknown	Beijing, China	Non-medicinal agarwood
C018	Chinese patent medicine B	unknown	Beijing, China	Mixed of non-medicinal agarwood
C019	Chinese patent medicine C	unknown	Haikou, China	Non-medicinal agarwood
C020	Chinese patent medicine D	unknown	Haikou, China	Medicinal agrwood/*A. sinensis*

### 2.2 DNA extraction and sequencing

Samples taken from leaves (100 mg) and dried wood or products (30 mg) were cleaned and rubbed for 5 min at a frequency of 30 r/s in a case of liquid nitrogen. DNA extraction proceeded using the modified HP plant DNA kit (OMEGA), with the addition of 1,000 μL of CPL and 10 μL of β-mercaptoethanol. The concentration and purity of the obtained sample DNA were determined, and the DNA was stored at −20°C for further use. Four batches of plant leaf DNA were interrupted using ultrasound; libraries were constructed by end repair, addition of sequencing junctions, purification, and PCR amplification; and the library fragment size and quality were examined. Sequencing was performed using Illumina’s high-throughput sequencing platform, NoVaSeq 6000.

### 2.3 Chloroplast genome assembly and annotation

Approximately 4 GB of raw data consisting of 150-bp paired-end reads were generated. Raw data in fastq format were first processed through fastp. In this step, clean data (clean reads) were obtained by removing reads containing the adapter, reads containing ploy-N, and low-quality reads from the raw data. At the same time, the content of guanine and cytosine (GC) was calculated. All downstream analyses were based on clean, high-quality data. Chloroplast genomes were assembled using GetOrganelle v1.7.6.1 with default settings, which filtered plastid-like reads, conducted *de novo* assembly, purified the assembly graph, and generated the complete chloroplast. All chloroplasts were initially annotated using PGA and GeSeq, with the annotated chloroplast of *A. subintegra* (NC052859) selected as the reference. For confirmation, all annotations were compared with previously published chloroplasts of *A. subintegra* (NC052859) and manually examined. Circular genome maps were visualized using OGDRAW v1.3.132. The sequences were aligned using MAFFT v7 with default settings (strategy of FFT-NS-2).

### 2.4 Development of species DNA mini-barcoding and HRM analysis

In this study, we compared and analyzed the chloroplast genomes of two medicinal agarwood species (*A. sinensis* and *A. agallocha)* and two non-medicinal species (*A. crassna* and *A. subintegra*) obtained by sequencing. The GenBank numbers of the four species are OR608759, OR608758, OR608757, and OR608760, respectively. Meanwhile, the chloroplast genomes of 12 published species of *Aquilaria* Lam. were selected in this study, including *A. agallocha* (NC065040 and MZ145047), *A. sinensis* (MN720647 and MN147870), *A. beccariana* (MN125347 and NC052855), *A. crassna* (MN125348 and NC043844), *A. cumingiana* (MZ145048 and NC065041), *A. hirta* (MN125349 and NC052856), *A. malaccensis* (MH286934 and NC041117), *A. microcarpa* (MN125350 and NC052858), *A. rostrata* (MN125351 and NC052858), *A. rugosa* (MZ145049 and NC065042), *A. subintegra* (MN147871 and NC052859), and *A. yunnanensis* (MG656407 and NC052859); full-length comparisons of these chloroplast genomes were performed using MEGA11 software; and the results were used to further screen for loci that could be used in medicinal agarwood, as well as other species-specific loci. Based on the specific loci, DNA mini-barcoding was designed for the identification of medicinal agarwood species using Oligo software. Primers that were likely to have dimers, hairpin structures, or excessive annealing temperatures were abandoned using Oligo Calc software.

PCR amplification, DNA melting, and end-point fluorescence level acquiring PCR amplifications were performed in a total volume of 20 μL on a Light Cycler^®^ 96 System (Roche). The reaction mixture contained 1 μL of genomic DNA, 10 μL of 2× *TransStart^®^* Tip Green qPCR SuperMix (Trans), 1 μL of 10 mM forward and reverse primers (**Table 2**), and nuclease-free water up to the final volume. The amplification protocol consisted of 10 min of pre-incubation at 95°C followed by 45 cycles under the following conditions: denaturing step at 95°C for 10 s, annealing at 58°C for 15 s, and extension at 72°C for 15 s. After the amplification, before the HRM step, the products were heated to 95°C for 60 s and then cooled to 40°C for 60 s. The HRM conditions were 65°C–95°C, rising to 0.07°C/s with 15 acquisitions per °C. In addition, these amplified PCR products were sequenced to verify the reliability of the results. All amplified products were sequenced by Guangzhou Ige Biotech Co., Ltd. The sequence was assembled using Codon Code Aligner 5.1.5 and aligned using ClustalW and DNAMAN.

**Table 2:** The primers were designed from loci in the chloroplast genome for mini-barcoding HRM analysis and identification.

Location	Primer name	Nucleotide sequence (5′ to 3′)	Product length	Identified species
*psb*M-*trn*D	Pt197F	GATTCACCGTCGAGAA	111 bp	*A. sinensis*
	Pt197R	ATCAATAAAGGAAACGAA		
*psb*A	1535F	AAGGCAATAATGAATACGGAA	148 bp	
	1535R	CAATTTTAGAGAGACGCGAAA		
*rps*16	5987F	CACGATAACCTTTTGAT	79 bp	
	5987R	GACTCAAAGCATTTTCAA		
*pet*N	30515F	TAGGGGACATAATTCACA	109 bp	
	30515R	TTCTTCCCCATACTACGA		
*ndh*E-*psa*C	125057F	AAATCAAAGTATCTTAGCCAC	87 bp	
	125057R	CGCCAGATGAATCAACGA		
*rps*4	48959F	ATTTAAGTAATTGTCGCTCT	89 bp	*A. agallocha*
	48959R	CCCGGAAAAACTCTCAA		
*atp*E	55861F	TCTGAGAGCTAGATTTGCCT	82 bp	
	55861R	GCAAACTCTTAAAATAGCGGAA		
*ycf*1	118649F	GGTATCGAGAAAAAGAAACT	89 bp	
	118649R	CAATATCGATTCATACAAGC		
*rps*15-*trn*N	148002F	AGTATGTATGACCATCGAG	80 bp	
	148002R	TCCAATGATCAAAGAAGTAGA		
*mat*K	M856F2	ATAGGATTGGTTACGGA	173 bp	*A. sinensis* *A. agallocha*
	M856R1	TTTTGCATTTATTACGGTTT		

To evaluate the repeatability of the HRM analysis method, the *A. sinensis*, *A. agallocha*, *A. crassna*, and *A. subintegra* species were randomly selected to perform the PCR reaction and HRM procedure, and the assays were performed in three repetitions. The parameters of the DNA template amount were tested using 1 ng, 5 ng, 10 ng, 25 ng, 50 ng, and 100 ng of DNA template in the reaction to evaluate the variation in the amount of DNA template. After obtaining suitable primers to test the sensitivity of the developed method, HRM was performed on standard samples. The *A. sinensis*, *A. agallocha*, and *A. crassna* sample powders were mixed in pairs at different ratios of 1%, 5%, 10%, 25%, 50%, 75%, 90%, 95%, and 99%. Real-time PCR amplification was performed as described earlier.

## 3 Results

### 3.1 Chlorplast genome characteristics of medicinal agarwood

**Figure 1:**
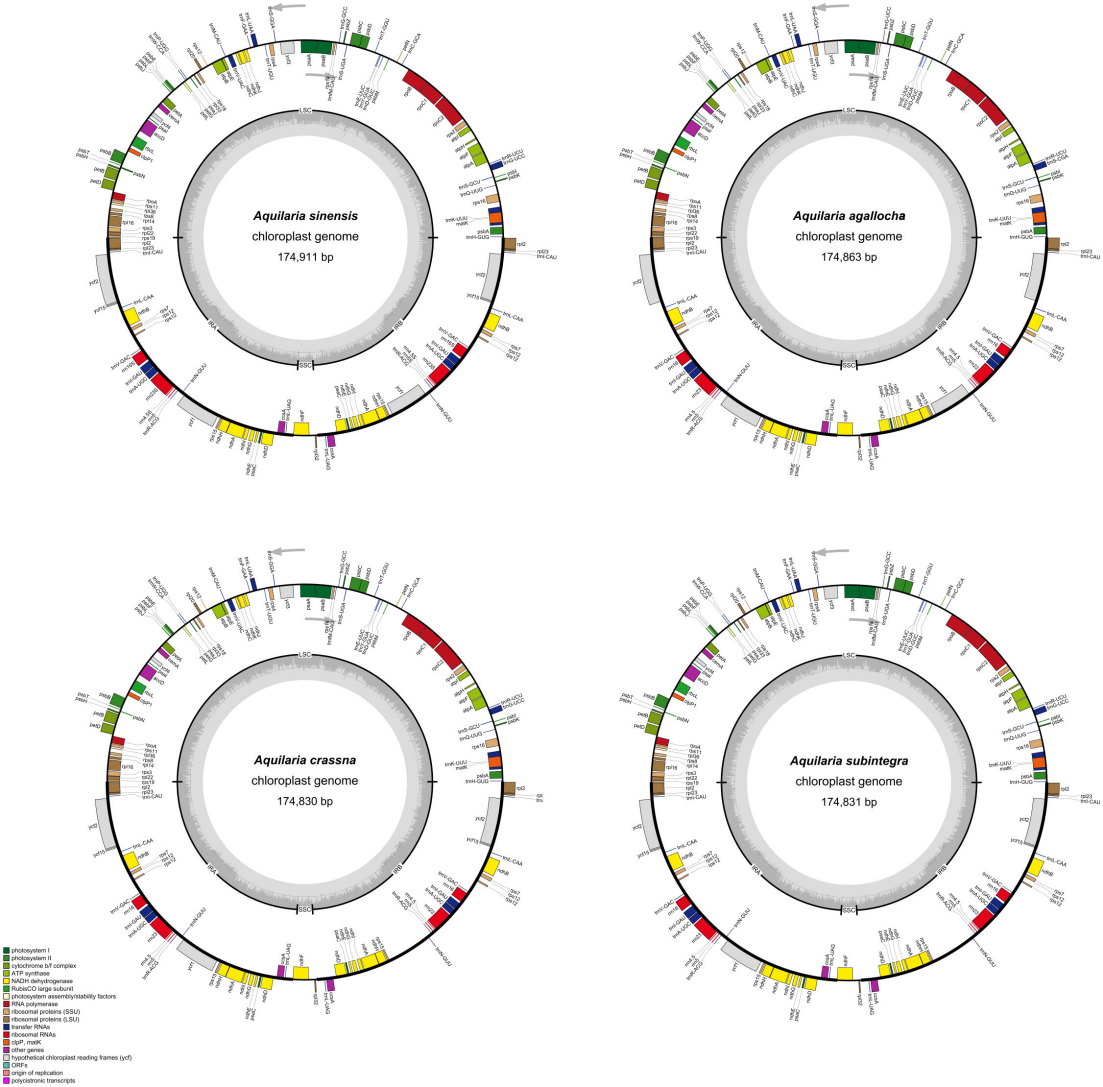
Four species gene map of the complete chloroplast genomes of *Aquilaria* Lam. in this study. The gray arrows represent the direction of gene transcription. The dark and light gray in the inner circle correspond to GC content and AT content, respectively.

For *A. sinensis*, *A. agallocha*, *A. crassna*, and *A. subintegra*, the total length of the circular chloroplast genome was determined to be 174,828–174,911 bp, containing a long single-copy sequence (LSC) region of 87,281–87,359 bp and a short single-copy sequence (SSC) region of 3,345–3,355 bp, separated by two SSCs (IRa and IRb) of 42,102–42,109 bp (**Figure 1**). The GC content of the four chloroplast genomes was 36.7%, with inverted repeat (IR) regions having a higher GC content (38.6%–38.8%) than LSC (34.9%–35.0%) and SSC (29.0%–29.1%). This differs from the published *A. agallocha* (MZ145047 and NC065040) and *A. subintegra* (MN147871 and NC052859) genomes by only 3 bp, whereas the GC contents of the four sequenced species are the same as those of the above published species. The difference between the sequenced species and *A. sinensis* (MN720647 and MN147870) is also only 3 bp or 4 bp, mainly in the LSC region, whereas the GC contents are the same. *A. crassna* (NC043844) exhibits differences relative to the sequenced species in the length of the IR and LSC regions (**Supplementary Table S1**). The IR/LSC and IR/SSC barcodes of the *A. sinensis*, *A. agallocha*, *A. crassna*, and *A. subintegra* chloroplast genomes were compared (**Supplementary Figure S1**). Among the four chloroplast genomes, the *rps*19 gene crossed the LSC and IRa regions with a 16-bp extension into the IRa region. The *ndh*F gene crossed the IRa/SSC boundary, with 28 bp, 27 bp, 26 bp, and 26 bp of extension into the IRa of *A. sinensis*, *A. agallocha*, *A. crassna*, and *A. subintegra*, respectively. The *rpl*2 gene was in the IRa and IRb regions, the *rpl*32 gene was in the SSC region, and the *psb*A and *rpl*22 genes were in the LSC region.

The four chloroplast genomes encode 141–142 genes, with the main difference being the *ycf*15 or *ycf*1 gene in the IR region. These genes include 95, 96, and 97 coding sequence, 38 transfer RNA genes, and eight ribosomal RNA genes. The first major group of genes related to transcription and translation includes ribosomal protein subunit genes, RNA polymerase genes, ribosomal RNA genes, and transfer RNA genes, totaling 77 genes, of which tRNA genes have the largest number, totaling 38; the second major group of genes related to photosynthesis includes photosystem I genes, photosystem II genes, nicotinamide adenine dinucleotide phosphate (NADPH) dehydrogenase genes, cytochrome b/f complex genes, ATP synthase, and rubisco large subunit. The third major group of genes related to other biosynthesis processes consisted of 14 genes (**Supplementary Table S2**).

The nucleotide diversity was estimated using the four chloroplast genomes of *Aquilaria* Lam., and the nucleotide diversity values (Pi) ranged from 0 to 0.02056, with an average of 0.001164, indicating that there is generally mild divergence among the genomes of these four species. However, as shown in **Supplementary Figure S2**, seven loci showed significantly higher Pi values (> 0.008); these included *ndh*C-*trn*V (0.02056), *psb*M-*trn*D (0.01289), *rps*4 (0.012), *psb*J-*pet*A (0.01144), *trn*k-*rps*16 (0.00911), *psa*A-*ycf*3 (0.00911), and *ndh*F-*rpl*32 (0.008), which can be applied to the mini-barcoding design.

### 3.2 Design and screening of mini-barcoding for traditional Chinese medicinal agarwood

In this study, by performing full-length comparisons of the chloroplast genomes of 12 species of the genus *Aquilaria* Lam. and combining the highly variable regions in the four chloroplast genomes obtained by sequencing, 59 SNP loci were identified that could distinguish two medicinal agarwood species (*A. sinensis* and *A. agallocha*) from 10 non-medicinal agarwood species, among which, 18 loci could distinguish *A. sinensis* from other species and 38 loci could distinguish *A. agallocha*. The other three loci could distinguish between medicinal agarwood and other species, but loci 130573 and 130574 needed to be used in combination thymine and adenine (TA) for medicinal agarwood and thymine and guanine (TG), guanine and guanine (GG), and guanine and adenine (GA) for other species) (**Supplementary Table S3**).

We designed 57 primer pairs (not listed) and obtained 10 improved primer pairs by qPCR screening. Five primer pairs obtained from the *psb*M*-trn*D, *psb*A, *rps*16, *pet*N, and *ndh*E-*psa*C regions could be used to distinguish *A. sinensis* from other species. Four primer pairs obtained from the *rps*4, *atp*E, *ycf*1, and *rps*15-*trn*N regions could be used to distinguish *A. agallocha* from other species. In addition, the primer pairs obtained from the *mat*K regions could distinguish between medicinal agarwood and other species.

**Figure 2:**
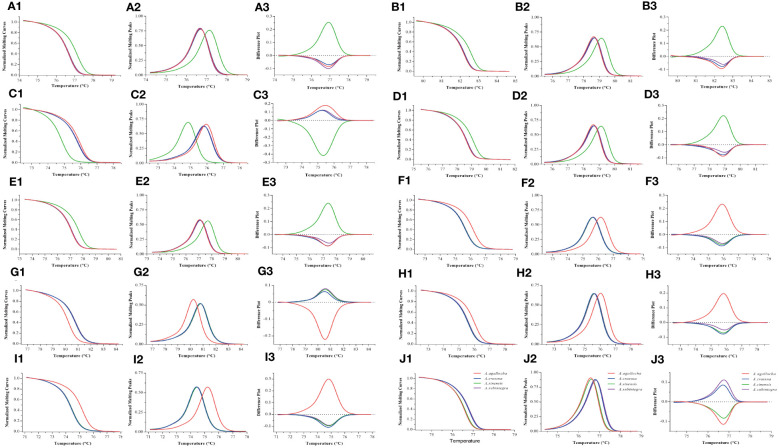
HRM melting curves for the identification of medicinal agarwood species with 10 pairs of primers. **(A–J)** Melting curve plots for primers pt197FR, 1535FR, 5987FR, 30515FR, 125057FR, 48959FR, 55861FR, 118649FR, 148002FR, and M856F2R1, respectively. 1–3 indicate three types of melting curves (normalized fluorescence curve, normalized fluorescence difference curve, and negative derivative of fluorescence versus temperature curve) for the same primer pair. The different colored lines represent the four types of agarwood (*A. sinensis*, *A. agallocha*, *A. crassna*, and *A. subintegra*).

We performed HRM analysis on four species of the genus *Aquilaria* (*A. sinensis*, *A. agallocha*, *A. crassna*, and *A. subintegra*) and screened 10 pairs of primers with the ability to identify medicinal and non-medicinal agarwood by plotting three melting curves. In this study, the normalized fluorescence curve (**Figures 2A1–J1**) and normalized fluorescence difference curve (**Figures 2A2–J2**) produced only different graphs. The normalized fluorescence curve demonstrates consistent results in the five primer pairs of the chloroplast genome (Pt197FR, 1535FR, 5987FR, 30515FR, and 125057FR), which could clearly identify *A. sinensis* from *A. agallocha*, *A. crassna*, and *A. subintegra* (**Figures 2A1–E1, A2–E2**). The four primers pairs (48959FR, 55861FR, 118649FR, and 148002FR) clearly identified *A. agallocha* from *A. sinensis*, *A. crassna*, and *A. subintegra* (**Figures 2F1–I1, F2–I2**). In addition, the primer pairs (M856F2R1) could simultaneously distinguish medicinal agarwood (*A. sinensis* and *A. agallocha*) from non-medicinal agarwood (*A. crassna* and *A. subintegra*) (**Figures 2J1, J2**). To intuitively describe the curve characteristics, we constructed the negative derivative of the fluorescence versus temperature (dF/dT) curve to show the melting temperature (Tm) of different samples (**Figures 2A3–J3**). The melting curve further magnifies the difference, making it easier to observe the difference between the four species. In short, the differences in these curves are caused by single-base variations in the sequence.

**Figure 3:**
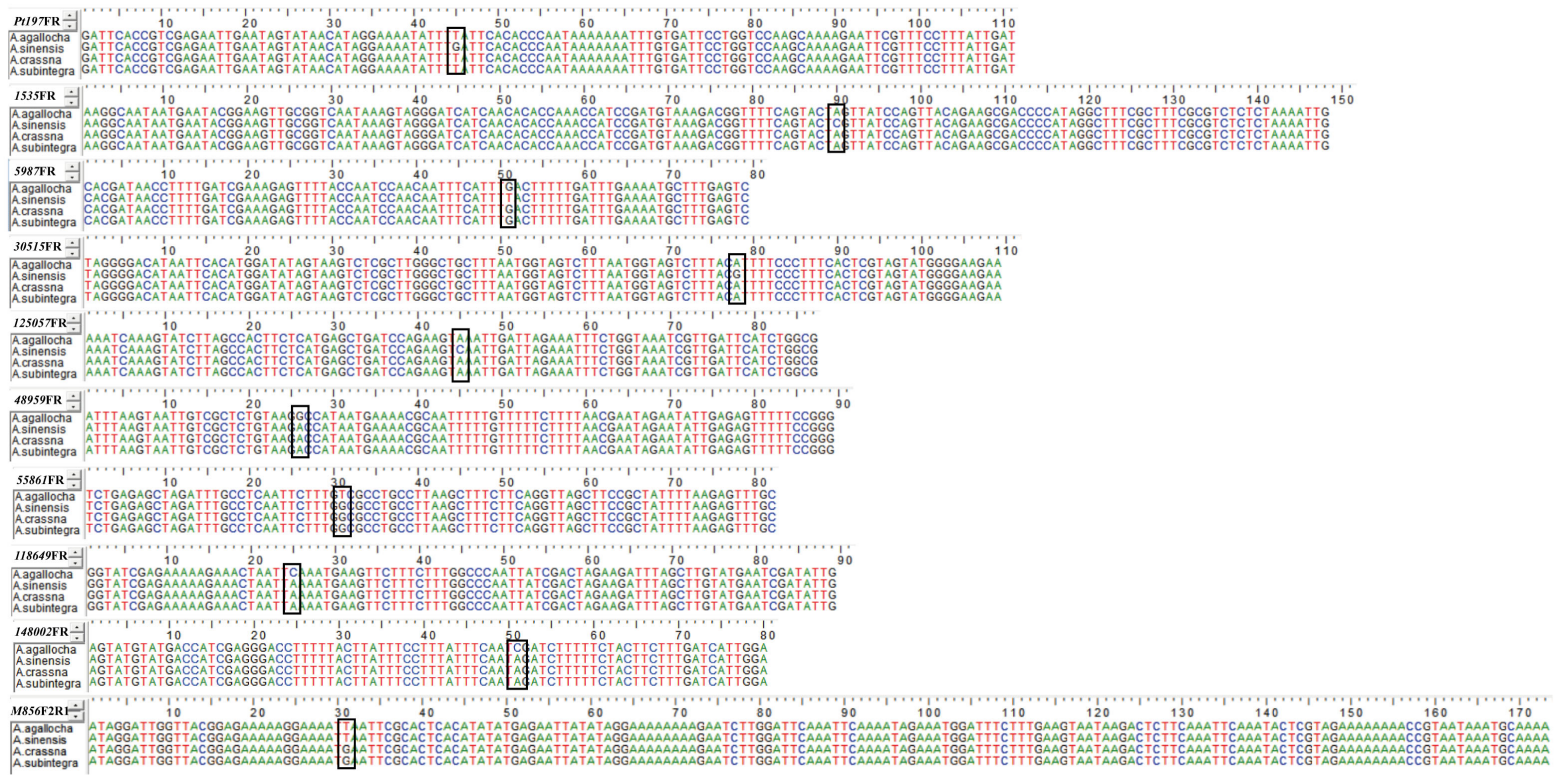
Sequencing results of PCR of 10 primer pairs for medicinal and non-medicinal agarwood. SNP loci are marked by black frame.

We sequenced and analyzed the PCR products of four species (*A. agallocha*, *A. sinensis*, *A. crassna*, and *A. subintegra*) amplified by 10 primer pairs, and the results were identical to the bases of the SNP loci (**Figure 3**), verifying that the differences in HRM solubility temperature and curve were due to differences in the bases of the SNP loci, indicating that the method was feasible.

### 3.3 Validation parameters for mini-barcoding HRM

**Figure 4:**
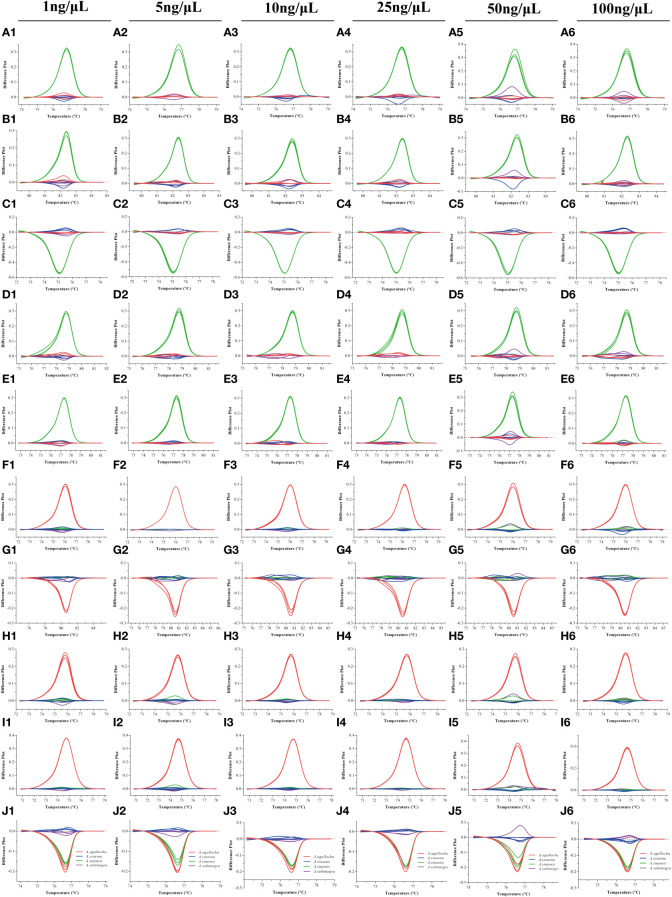
Fluorescence difference curve for changes in the amount of DNA templates in *A. agallocha*, *A. sinensis*, *A. crassna*, and *A. subintegra*. The range of 1 ng, 5 ng, 10 ng, 25 ng, 50 ng, and 100 ng of DNA template per 20 μL of reaction was tested. HRM analysis results of each primer pair: Pt197FR **(A1–A6)**, 1535FR **(B1–B6)**, 5987FR **(C1–C6)**, 30515FR **(D1–D6)**, 125057FR **(E1–E6)**, 48959FR **(F1–F6)**, 55861FR **(G1–G6)**, 118649FR **(H1–H6)**, 148002FR **(I1–I6)**, and M856F2R1 **(J1–J6)**.

In this study, we investigated the amount of template DNA and the melting curve. The normalized fluorescence difference curve results (**Figure 4**) demonstrated that the correct groupings were obtained in the range of 1 ng to 100 ng of DNA for the four *Aquilaria* Lam. species with 10 primer pairs. The melting curve of the non-medicinal agarwood slightly deviated only when 50 ng of template DNA was used, but this did not affect the identification and classification of the species. In the applied HRM analysis, the melting curves were more stable at low template concentrations (≤ 25 ng/μL) than at high concentrations (≥ 50 ng/μL) within an appropriate dynamic range. We statistically analyzed the melting temperatures for the six DNA template concentrations (1 ng/μL to 100 ng/μL) from four agarwood species (**Table 3**).

**Table 3:** HRM melting temperature (Tm) values of medicinal agarwood for 10 pairs of primers.

Primer	Melting temperature (Tm)/°C			
	Medicinal agarwood		Non-medicinal agarwood	
	*A. agallocha*	*A. sinensis*	*A. crassna*	*A. subintegra*
Pt197FR	76.56–76.63 (76.59 ± 0.02)	**76.96–77.09 ^#^** **(77.02 ± 0.03)**	76.56–76.65 (76.59 ± 0.03)	76.52–76.69 (76.60 ± 0.04)
1535FR	82.05–82.23 (82.14 ± 0.04)	**82.46–82.58** **(82.54 ± 0.02)**	82.07–82.23 (82.15 ± 0.04)	82.12–82.23 (82.15 ± 0.02)
5987FR	75.58–75.71 (75.62 ± 0.04)	**74.53–74.62** **(74.57 ± 0.03)**	75.52–75.58 (75.54 ± 0.02)	75.51–75.61 (75.54 ± 0.03)
30515FR	78.47–78.54 (78.49 ± 0.02)	**78.95–79.02** **(78.98 ± 0.03)**	78.47–78.56 (78.51 ± 0.04)	78.47–78.55 (78.50 ± 0.03)
125057FR	76.98–77.05 (77.00 ± 0.02)	**77.57–77.65** **(77.61 ± 0.02)**	76.98–77.07 (77.01 ± 0.03)	76.98–77.06 (77.01 ± 0.03)
48959FR	**76.26–76.32** **(76.29 ± 0.02)**	75.68–75.81 (75.76 ± 0.03)	75.68–75.79 (75.74 ± 0.04)	75.73–75.80 (75.75 ± 0.02)
55861FR	**80.21–80.31** **(80.26 ± 0.03)**	80.80–80.84 (80.80 ± 0.03)	80.80–80.84 (80.78 ± 0.05)	80.73–80.84 (80.77 ± 0.03)
118649FR	**75.93–76.04** **(75.99 ± 0.03)**	75.51–75.59 (75.55 ± 0.03)	75.47–75.59 (75.54 ± 0.03)	75.51–75.57 (75.54 ± 0.02)
148002FR	**75.03–75.10** **(75.06 ± 0.03)**	74.31–74.38 (74.34 ± 0.03)	74.27–74.40 (74.34 ± 0.04)	74.31–74.38 (74.34 ± 0.03)
M856F2R1	**76.44–76.53** **(76.48 ± 0.03)**	**76.46–76.58** **(76.51 ± 0.03)**	76.64–76.77 (76.70 ± 0.04)	76.64–76.73 (76.69 ± 0.03)

^#^Marked in bold are the melting temperatures that distinguish the species of the target medicinal agarwood.

Different primers produced different melting temperatures for the target to distinguish the species of medicinal agarwood. Among them, the melting temperatures of five primer pairs (Pt197FR, 1535FR, 5987FR, 30515FR, and 125057FR) could distinguish *A. sinensis* from other species; four primer pairs (48959FR, 55861FR, 118649FR, and 148002FR) could distinguish *A. agallocha* from other species; and primer M856F2R1 could distinguish two medicinal agarwood species (*A. sinensis* and *A. agallocha*) from other agarwood species.

**Figure 5:**
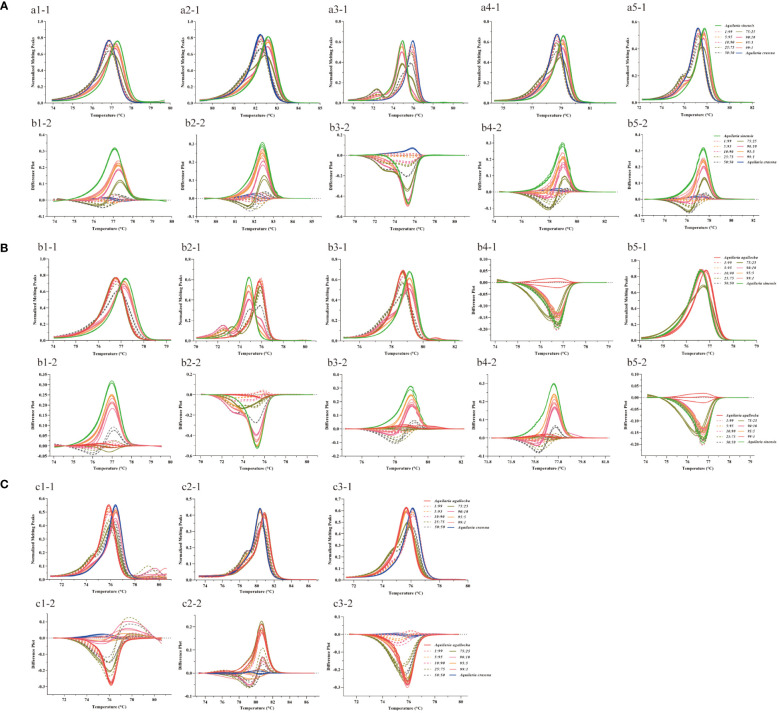
Melting curves of mixed samples of medicinal and non-medicinal agarwood analyzed by mini-barcode HRM. The different colored lines represent the ratios for mixing agarwood (1%, 5%, 10%, 25%, 50%, 75%, 90%, 95%, and 99%). **(A–C)** Mixing *A. sinensis* with *A. crassna*, *A. sinensis* with *A. agallocha*, and *A. agallocha* with *A. crassna* samples, respectively. a1-a5: Melting curves of primers Pt197FR, 1535FR, 5987FR, 30515FR, and 125057FR; b1-b5: Melting curves of primers Pt197FR, 5987FR, 30515FR, 125057FR, and M856-F2R1; c1-c5: Melting curves of primers 48959FR, 55861FR, and 118649FR; 1-1, 2-1, 3-1, 4-1, and 5-1 are the normalized fluorescence difference curves; 1-2, 2-2, 3-2, 4-2, and 5-2 are the negative derivatives of the fluorescence versus temperature curves.

We first extracted DNA by mixing different agarwood powders in proportion to each other and then verified the adulteration sensitivity of medicinal and non-medicinal agarwood using the mini-barcoding HRM method. We mixed the samples of *A. sinensis*, *A. agallocha*, and *A. crassna* with each other and obtained the normalized fluorescence difference curves and the negative derivatives of the fluorescence versus temperature curves by HRM analysis to determine the results. When the primers Pt197FR, 1535FR, 5987FR, 30515FR, and 125057FR were used for the adulteration verification of *A. sinensis* and *A. crassna* (**Figure 5A**), the melting curve line shape of the mixed samples deviated from that of the homozygote when other agarwood samples were added to the domestic medicinal agarwood (*A. sinensis*) was more obvious in the identification of the negative derivative of the fluorescence versus temperature curve. Similarly, using primers Pt197FR, 5987FR, 30515FR, 125057FR, and M856-F2R1, the mixed adulteration verification of domestic medicinal agarwood with imported medicinal agarwood revealed that the line shape of the melting curves of the mixed samples also deviated from those of the homozygote samples (**Figure 5B**). The melting curve of the HRM mixed sample using primer 5987FR showed an extra peak (**Figures 5A**a3–1, **B**b2–1). Additionally, the adulteration of imported medicinal agarwood (*A. agallocha*) was verified using primers 48959FR, 55861FR, and 118649FR, and the line shape of the melting curve changed as the amount of other agarwood increased (**Figure 5C**). The detection limit of adulteration in medicinal agarwood was examined by this method, and the melting curve line shape at ≥ 1% of adulteration was compared to that of the homozygote. However, as a control, the addition of a homozygote of medicinal agarwood is essential in HRM analysis. Therefore, this method can effectively determine whether the samples are adulterated or not.

### 3.4 Identification of traditional Chinese medicinal agarwood species in commercial products

In this study, HRM analysis and PCR sequencing of 20 samples using 10 primer pairs gave consistent results. Eight batches of samples were medicinal agarwood (including one batch of proprietary Chinese medicines containing agarwood), and 11 batches were non-medicinal agarwood (including two batches of proprietary Chinese medicines containing agarwood). One batch of proprietary Chinese medicines containing agarwood may have used medicinal agarwood mixed with non-medicinal agarwood (adulteration rate between 5% and 10%), but this requires further verification at a later stage. Of these, seven batches (C001, C002, C003, C004, C011, C016, and C020) of medicinal agarwood samples were *A. sinensis*, and one batch (C014) was *A. agallocha*. Most of the 11 batches of non-medicinal agarwood originated from Vietnam, Malaysia, Thailand, Kalimantan, Papua New Guinea, and other Southeast Asian countries (**Table 1**; **Supplementary Table S4**). Although no further species identification of these 11 batches of non-medicinal agarwood samples was performed, this does not affect the judgment of the results. The 10 primer pairs generated in this study were effective in identifying the species of medicinal agarwood samples, among which primer M856-F2R1 could directly and rapidly determine whether the species of the samples were medicinal agarwood. Nevertheless, two specific sites were simultaneously present in samples C018 (primers 30515FR, 125057FR, and M856F2R1) and C019 (primers 1535FR and 118649FR) when amplified sequences from those samples were sequenced. Furthermore, the amplified primer 5987FR sequence from the C019 sample showed heterozygous sequencing. Future research will shed more light on these unanticipated occurrences.

## 4 Discussion

### 4.1 Importance of traditional Chinese medicinal agarwood species identification

It has been reported that both species of the genus *Aquilaria* and *Gyrinops* (Thymelaeaceae) can produce agarwood (Lee et al., 2022), a resin-containing wood (Liu et al., 2013, 2019), and it is difficult to differentiate the species information during the trade process, especially when it is crushed and added to preparations to improve the ease of adulteration. Currently, there are only two legal species of traditional Chinese medicinal agarwood in China. Given the high use of commercially available agarwood and the fact that approximately 138 types of medicines contain agarwood, it is meaningful to develop a method to rapidly and accurately identify the species of Chinese medicinal agarwood. Our findings will provide a reference for the identification of medicinal agarwood species in Japan and the Taiwan Province of China, which is of practical significance and application value for the development of the agarwood pharmaceutical industry.

Until now, molecular identification methods for agarwood have mainly focused on DNA barcode screening and its combinations, such as *ITS*2+t*rn*L-*trn*F (Lee et al., 2016), *ITS*2+*mat*K (Kang et al., 2018), *ITS*+*mat*K, *ITS*+*rbc*L, *ITS*+*trn*L-*trn*F (Li et al., 2018; Lin et al., 2023), and *psb*J-*pet*A+*trn*T-*trn*L (Hishamuddin et al., 2023). However, all of these methods require agarwood plant material as the basis for the identification of species using sequencing and construction of phylogenetic trees. In addition, these barcode amplification products require long lengths, such as the *ITS* length of approximately 673 bp, the *mat*K length of approximately 778 bp, and the *trn*L-*trn*F length of approximately 938 bp or even longer. Moreover, as DNA extracted from agarwood is stored for a long time and can undergo severe degradation, the amplification results using these primers are often unsuccessful. Consequently, plant leaves are often required to build DNA barcode data combinations or databases, which is difficult to apply in practice. Therefore, in this study, by comparing the chloroplast genome sequences of different species of the genus *Aquilaria* Lam., we screened for SNP sites and used the combination of mini-barcoding and HRM, which can either visually determine the species from the melting curve or sequence the amplification products to obtain the specific base sites. This method avoids the need to construct multiple DNA barcode combination libraries, which improves the timeliness and accuracy of traditional Chinese medicinal agarwood species screening.

### 4.2 Establishment and validation of mini-barcoding HRM methods

SNP molecular marker technology can be used to identify abundant loci and has been applied to the analysis of germplasm resource evolution and species identification (Li et al., 2023; Palasciano et al., 2023). Chloroplasts are maternally inherited, and the genetic information of each species can be stably inherited. By analyzing the differences in SNP and designing the corresponding primers, it is possible to accurately identify different species. In the present study, 59 SNP loci were obtained from the chloroplast genomes of 12 published species of *Aquilaria* Lam. and could be used to distinguish traditional Chinese medicinal agarwood (*A. sinensis* and *A. agallocha*) from non-medicinal agarwood species. The SNP loci were obtained correctly along with the four agarwood chloroplast genomes obtained by sequencing in this study. We further screened to obtain the 10 best primer pairs, of which five pairs could distinguish *A. sinensis* from other species, four pairs could distinguish *A. agallocha* from other species, and another one pair could distinguish two medicinal agarwood species from non-medicinal agarwood. These primers were from the *psb*M-*trn*D, *psb*A, *rps*16, *pet*N, *ndh*E-*psa*C, *rps*4, *atp*E, *ycf*1, *rps*15-*trn*N, and *mat*K regions, and their amplification products were < 200 bp with high amplification success.

HRM analysis is a simple, rapid, and cost-effective method for identifying genetic variants. The dissociation of double-stranded DNA is measured in real time by HRM, and the polymorphism of the sample DNA can be easily monitored according to the shape and position of the melting curve (Gundry et al., 2003; Vossen et al., 2009; Suesatpanit et al., 2017; Kim et al., 2023). In this study, we successfully screened the chloroplast genome from the genus *Aquilari*a Lam. for species identification of SNP loci that can be used for medicinal agarwood and validated them by applying HRM and sequencing. We conducted methodological investigations using these 10 primer pairs, and the melting curve line shapes and melting temperatures were correctly grouped for agarwood sample DNA concentrations in the range of 1 ng to 100 ng. In the applied HRM analysis, the melting curves were more stable at low template concentrations (≤ 25 ng/μL). We also examined the detection limits of adulteration using different ratios of agarwood powder. The melting curves and temperatures deviated from those of the homozygote sample after the addition of non-medicinal agarwood to the medicinal agarwood samples, and the detection limit of adulteration was 1%. The method was validated in 20 batches of commercially available samples, among which eight batches were identified as medicinal agarwood (seven batches were traditional Chinese medicinal agarwood and one batch was imported medicinal agarwood), and 11 batches were non-medicinal agarwood. Additionally, we unexpectedly found that one batch of proprietary Chinese medicine containing agarwood used a mixture of medicinal and non-medicinal agarwood, with an adulteration rate between 5% and 10%, although this needs to be further verified at a later stage.

However, it is usually during HRM analysis that false-positive results are most likely to occur. The main practices that are effective in avoiding false positives are the use of multiple characterized SNP loci assessment, amplified sequence sequencing validation, and the use of target species controls. In addition, the occurrence of false-positive results is also related to PCR amplification conditions and target DNA concentration and purity. In this study, the above situation was evaluated, and the 10 pairs of primers obtained in this study can satisfy the identification of two medicinal agarwood species, and, according to the purpose, one or more of the primers can be selected to carry out species identification. If necessary, the reliability of the method can be verified by direct sequencing of the amplification products, which, in this study, was shown to be reliable by the sequencing results. In addition, methodological validation was performed in this study, and because of the differences in DNA concentration and purity between different samples and the reaction reagents themselves, we suggest that, when using this method, it will be necessary to add positive homozygote samples for analysis.

Therefore, compared to the traditional DNA barcode analysis method, the method established in this study is time-sensitive and can be used to quickly, intuitively, and accurately determine the medicinal agarwood species. In the future, in addition to the traditional Chinese medicine agarwood species identified in this study, there are many other agarwood species that need to be identified, and the mini-barcoding of each species can be further developed along the lines of this study according to the usage or industry needs.

## 5 Conclusion

In this study, based on chloroplast genomic data, we established an identification method for medicinal agarwood using mini-barcoding combined with HRM and designed and validated 10 pairs of primers from the *psb*M-*trn*D, *psb*A, *rps*16, *pet*N, *ndh*E-*psa*C, *rps*4, *atp*E, *ycf*1, *rps*15-*trn*N, and *mat*K regions. The amplification products were all < 200 bp, with a high success rate of amplification. The method was applied to successfully identify traditional Chinese medicinal agarwood species from commercial agarwood samples. Overall, the sensitivity of this method was sufficient to detect 1% of adulterants in medicinal agarwood products, proving that mini-barcoding HRM is a powerful and flexible tool. This represents a fast and effective high-throughput method for authenticity testing of traditional Chinese medicinal agarwood and its raw materials containing agarwood-containing proprietary Chinese medicines and is recommended for industrial applications.

## Data availability statement

The data presented in the study are deposited in the Genbank repository, accession number OR608757 - OR608760. The original contributions presented in the study are included in the article/supplementary material, further inquiries can be directed to the corresponding author.

## Author contributions

JF: Writing – review & editing, Writing – original draft, Software, Methodology, Investigation, Formal analysis, Data curation. YL: Writing – original draft, Writing – review & editing, Funding acquisition, Conceptualization. AX: Writing – review & editing, Data curation. YY: Writing – review & editing, Investigation. FL: Writing – review & editing, Visualization. JW: Writing – review & editing, Funding acquisition, Conceptualization.
