# Supplementary material for: Successful development of molecular diagnostic technology combining mini-barcoding and high-resolution melting for traditional Chinese medicine agarwood species based on single-nucleotide polymorphism in the chloroplast genome
Source: Front Plant Sci. 2024 Jul 31;15:1405168. doi: 10.3389/fpls.2024.1405168 (PMC11322813; doi:10.3389/fpls.2024.1405168)
Supplement: Supplementary file 1 [file DataSheet_1.zip › Tables S1 - S4.docx]

Supplementary Material

# Supplementary Tables

**Table S1** The complete chloroplast genome, IR, LSC, and SSC lengths (bp), and GC content of four species of chloroplast genomes of *Aquilaria* Lam.

| Species | *A. sinensis* | | | *A. agallocha* | | |
| --- | --- | --- | --- | --- | --- | --- |
| Genbank no | MN720647 | MN147870 | OR608759 | NC065040 | MZ145047 | OR608758 |
| Genome size (bp) | 174,914 | 174,907 | 174,911 | 174,866 | 174,866 | 174,863 |
| GC content (%) | 36.7 | 36.7 | 36.7 | 36.7 | 36.7 | 36.7 |
| IR length (bp) | 42,103 | 42,103 | 42,103 | 42,108 | 42,108 | 42,109 |
| GC content in IR (%) | 38.9 | 38.9 | 38.9 | 38.8 | 38.8 | 38.8 |
| LSC length (bp) | 87,361 | 87,355 | 87,359 | 87,294 | 87,294 | 87,290 |
| GC content in LSC (%) | 34.9 | 34.9 | 34.9 | 35.0 | 35.0 | 35.0 |
| SSC length (bp) | 3,346 | 3,346 | 3,346 | 3,355 | 3,355 | 3,355 |
| GC content in SSC (%) | 29.0 | 29.1 | 29.1 | 29.0 | 29.0 | 29.0 |
| Date resource | Hishamuddin *et al.*, 2020 | Lee *et al.*, 2022 | This study | Lee *et al.*, 2022 | Lee *et al.*, 2022 | This study |
| Species | *A. crassna* | | | *A. subintegra* | | |
| Genbank no | MN125348 | NC043844 | OR608757 | MN147871 | NC052859 | OR608760 |
| Genome size (bp) | 174,830 | 174,830 | 174,830 | 174,828 | 174,828 | 174,831 |
| GC content (%) | 36.7 | 36.7 | 36.7 | 36.7 | 36.7 | 36.7 |
| IR length (bp) | 42,102 | 42,017 | 42,102 | 42,102 | \| 42,102 \| \| --- \| | 42,102 |
| GC content in IR (%) | 38.8 | 38.6 | 38.8 | 38.8 | 38.8 | 38.8 |
| LSC length (bp) | 87,281 | 87,450 | 87,281 | 87,279 | 87,279 | 87,281 |
| GC content in LSC (%) | 34.9 | 34.9 | 34.9 | 34.9 | 34.9 | 34.9 |
| SSC length (bp) | 3345 | 3,346 | 3,345 | 3,345 | 3,345 | 3,346 |
| GC content in SSC (%) | 29.1 | 29.1 | 29.1 | 29.1 | 29.1 | 29.1 |
| Date resource | Lee *et al.*, 2022 | Li *et al.*, 2019 | This study | Lee *et al.*, 2022 | Hishamuddin *et al.*, 2020 | This study |

**Table S2** Genetic composition of the chloroplast genomes of four species of chloroplast genomes of *Aquilaria* Lam.

| Gene function | Gene category | Gene name | Number |
| --- | --- | --- | --- |
| Self-replication | Large subunit of ribosome | *rpl2* ^(×2)^, *rpl14*, *rpl16*, *rpl20*, *rpl22*, *rpl23* ^(×2)^, *rpl32*, *rpl33*, *rpl36* | 11 |
|  | Small subunit of ribosome | *rps2*, *rps3*, *rps4*, *rps7* ^(×2)^, *rps8*, *rps11*, *rps12* ^(×3)^, *rps14*, *rps15* ^(×2)^, *rps16*, *rps18*, *rps19* | 16 |
|  | RNA polymerase | *rpoA*, *rpoB*, *rpoC1*, *rpoC2* | 4 |
|  | Ribosomal RNAs | *rrn16s* ^(×2)^, *rrn23s* ^(×2)^, *rrn4.5s* ^(×2)^, *rrn5s* ^(×2)^ | 8 |
|  | Transfer RNAs | *trnA-UGC* ^(×2)^, *trnC-GCA*, *trnD-GUC*, *trnE-UUC*, *trnF-GAA*, *trnfM-CAU*, *trnG-GCC*, *trnG-UCC*, *trnH-GUG*, *trnI-GAU* ^(×2)^, *trnI-CAU* ^(×2)^, *trnK-UUU*, *trnL-CAA* ^(×2)^, *trnL-UAA*, *trnL-UAG* ^(×2)^, *trnM-CAU*, *trnN-GUU* ^(×2)^, *trnP-UGG*, *trnQ-UUG*, *trnR-UCU*, *trnR-ACG* ^(×2)^, *trnS-GCU*, *trnS-UGA*, *trnS-GGA*, *trnT-UGU*, *trnT-GGU*, *trnV-GAC* ^(×2)^, *trnV-UAC*, *trnW-CCA*, *trnY-GUA* | 38 |
| Photosynthesis | Photosystem Ⅰ | *psaA*, *psaB*, *psaC* ^(×2)^, *psaI*, *psaJ* | 6 |
|  | Photosystem Ⅱ | *psbA*, *psbB*, *psbT*, *psbK*, *psbI*, *psbH*, *psbM*, *psbN*, *psbD*, *psbC*, *psbZ*, *psbJ*, *psbL*, *psbE*, *psbF* | 15 |
|  | NADPH dehydrogenase | *ndhA* ^(×2)^, *ndhB* ^(×2)^, *ndhC*, *ndhD* ^(×2)^, *ndhE* ^(×2)^, *ndhF*, *ndhH* ^(×2)^, *ndhG* ^(×2)^, *ndhJ*, *ndhK*, *ndhI* ^(×2)^ | 18 |
|  | Cytochrome b/f complex | *petA*, *petB*, *petD*, *petN*, *petL*, *petG* | 6 |
|  | ATP synthase | *atpA*, *atpB*, *atpE*, *atpF*, *atpH*, *atpI* | 6 |
|  | Rubisco large subunit | *rbcL* | 1 |
| Other genes | Maturase | *matK* | 1 |
|  | Membrane protein | *cemA* | 1 |
|  | Subunits of Acetyl-CoA-carboxylase | *accD* | 1 |
|  | C-type cytochrome synthesis gene | *ccsA* ^(×2)^ | 2 |
|  | Protease | *clpP* | 1 |
| Unknown gene | Hypothetical chloroplast reading frames | *ycf1^#^* ^(×2)^, *ycf15^*^* ^(×2)^, *ycf2* ^(×2)^, *ycf3*, *ycf4* | 8 |

^*^ For the IR region of the gene in the chloroplast genome of *A. sinensis*. ^#^ indicates that only one gene is present in *A. crassna* and *A. subintegra*.

**Table S3** SNP loci in the complete chloroplast genome of *Aquilaria* Lam.

| SNP  Positions | 1535 | 1795 | 2297 | 3074 | 3410 | 4233 | 5987 | 7465 | 9427 | 14456 | 15373 | 15891 | 30515 | 31140 | 31147 | 31967 | 33457 | 36979 | 38762 | 39187 | 45330 | 48959 | 49643 | 51898 | 54910 | 55198 | 55449 | 55861 | 56515 |
| --- | --- | --- | --- | --- | --- | --- | --- | --- | --- | --- | --- | --- | --- | --- | --- | --- | --- | --- | --- | --- | --- | --- | --- | --- | --- | --- | --- | --- | --- |
| *A. sinensis* | **C** | C | A | **T** | G | **A** | **T** | **G** | G | **A** | A | C | **G** | **C** | C | **G** | C | **T** | **C** | G | T | A | T | A | A | **G** | T | G | C |
| *A. agallocha* | A | **G** | **G** | **T** | **T** | C | G | T | **A** | G | **C** | **A** | A | A | **G** | T | **G** | C | A | **A** | **A** | **G** | **G** | **C** | **T** | C | **G** | **T** | **T** |
| *Other species^#^* | A | C | A | G | G | C | G | T | G | G | A | C | A | A | C | T | C | C | A | G | T | A | T | A | A | C | T | G | C |
| SNP  Positions | 61905 | 63678 | 64580 | 67155 | 67683 | 68460 | 70578 | 72267 | 72436 | 76927 | 78857 | 84767 | 85124 | 87416 | 90565 | 102786 | 115174 | 118649 | 122120 | 125057 | 130573 | 130574 | 138119 | 141056 | 144527 | 148002 | 160390 | 172611 |  |
| *A. sinensis* | G | **G** | **G** | T | T | T | C | T | C | T | **T** | **C** | **C** | **T** | G | G | T | A | C | **C** | **T** | **A** | **G** | G | T | A | C | C |  |
| *A. agallocha* | **C** | C | T | **G** | **G** | **G** | **T** | **A** | **G** | **G** | G | T | G | G | **A** | **T** | **G** | **C** | **T** | A | **T** | **A** | T | **A** | **G** | **C** | **A** | **T** |  |
| *Other species* | G | C | T | T | T | T | C | T | C | T | G | T | G | G | G | G | T | A | C | A | T/G | G/A | T | G | T | A | C | C |  |

^#^ The other species were 10 species of the genus *Aquilaria* Lam., including *A. beccariana* (MN125347 and NC052855), *A. crassna* (MN125348 and NC043844), *A. cumingiana* (MZ145048 and NC065041), *A. hirta* (MN125349 and NC052856), *A. malaccensis* (MH286934 and NC041117), *A. microcarpa* (MN125350 and NC052858), *A. rostrata* (MN125351 and NC052858), *A. rugosa* (MZ145049 and NC065042), *A. subintegra* (MN147871 and NC052859), and *A. yunnanensis* (MG656407 and NC036940).

**Table S4** Species identification results of commercially agarwood samples using mini-barcoding HRM.

| **Sample No.** | **Trade species** | **Result judgment** | | | **Species**  **identification result** |
| --- | --- | --- | --- | --- | --- |
|  |  | **primers** | **Sequencing specific base site** | **HRM**  **melting temperature** |  |
| C001 | *A.sinensis* | *Pt197FR*  *1535FR*  *5987FR*  *30515FR*  *125057FR*  *M856F21R* | G  C  T  G  C  T | 77.06±0.04  82.45±0.04  74.52±0.00  78.99±0.04  77.51±0.00  76.44±0.00 | *A. sinensis*  (Medicinal agarwood) |
| C002 | *A.sinensis* | *Pt197FR*  *1535FR*  *5987FR*  *30515FR*  *125057FR*  *M856F21R* | G  C  T  G  C  T | 77.04±0.00  82.47±0.04  74.52±0.00  79.01±0.07  77.53±0.01  76.44±0.00 | *A. sinensis*  (Medicinal agarwood) |
| C003 | unknown | *Pt197FR*  *1535FR*  *5987FR*  *30515FR*  *125057FR*  *M856F21R* | G  C  T  G  C  T | 77.06±0.04  82.47±0.04  74.58±0.00  78.90±0.04  77.58±0.00  76.48±0.04 | *A. sinensis*  (Medicinal agarwood) |
| C004 | unknown | *Pt197FR*  *1535FR*  *5987FR*  *30515FR*  *125057FR*  *M856F21R* | G  C  T  G  C  T | 77.04±0.00  82.49±0.00  74.56±0.04  78.86±0.00  77.58±0.00  76.51±0.00 | *A. sinensis*  (Medicinal agarwood) |
| C005 | unknown | *Pt197FR*  *1535FR*  *5987FR*  *30515FR*  *125057FR*  *48959FR*  *55861FR*  *118649FR*  *148002FR*  *M856F21R* | T  A  G  A  A  A  G  A  A  G | 76.56±0.04  82.06±0.04  75.44±0.00  78.49±0.00  76.92±0.00  75.62±0.04  80.87±0.04  75.46±0.00  74.24±0.04  76.66±0.00 | Non-medicinal agarwood |
| C006 | unknown | *Pt197FR*  *1535FR*  *5987FR*  *30515FR*  *125057FR*  *48959FR*  *55861FR*  *118649FR*  *148002FR*  *M856F21R* | T  A  G  A  A  A  G  A  A  G | 76.45±0.00  82.08±0.04  75.46±0.04  78.49±0.00  76.94±0.04  75.57±0.04  80.74±0.04  75.46±0.07  74.28±0.00  76.70±0.04 | Non-medicinal agarwood |
| C007 | unknown | *Pt197FR*  *1535FR*  *5987FR*  *30515FR*  *125057FR*  *48959FR*  *55861FR*  *118649FR*  *148002FR*  *M856F21R* | T  A  G  A  A  A  G  A  A  G | 76.00±0.00  82.08±0.04  75.44±0.00  78.51±0.04  76.92±0.00  75.59±0.00  80.79±0.04  75.46±0.00  74.21±0.00  76.75±0.04 | Non-medicinal agarwood |
| C008 | unknown | *Pt197FR*  *1535FR*  *5987FR*  *30515FR*  *125057FR*  *48959FR*  *55861FR*  *118649FR*  *148002FR*  *M856F21R* | T  A  G  A  A  A  G  A  A  G | 76.58±0.00  82.23±0.00  75.57±0.00  78.49±0.00  77.05±0.00  75.72±0.00  80.83±0.00  75.59±0.00  74.41±0.00  76.77±0.00 | Non-medicinal agarwood |
| C009 | unknown | *Pt197FR*  *1535FR*  *5987FR*  *30515FR*  *125057FR*  *48959FR*  55861FR  *118649FR*  *148002FR*  *M856F21R* | T  A  G  A  A  A  G  A  A  G | 76.54±0.04  82.08±0.04  75.46±0.04  78.51±0.04  76.99±0.00  75.59±0.00  80.87±0.04  75.50±0.04  74.28±0.00  77.05±0.00 | Non-medicinal agarwood |
| C010 | unknown | *Pt197FR*  *1535FR*  *5987FR*  *30515FR*  *125057FR*  *48959FR*  *55861FR*  *118649FR*  *148002FR*  *M856F21R* | T  A  G  A  A  A  G  A  A  G | 76.67±0.04  82.19±0.04  75.52±0.04  78.60±0.04  77.03±0.04  75.64±0.04  80.79±0.04  75.59±0.00  74.35±0.00  76.66±0.07 | Non-medicinal agarwood |
| C011 | unknown | *Pt197FR*  *1535FR*  *5987FR*  *30515FR*  *125057FR*  *M856F21R* | G  C  T  G  C  T | 77.01±0.04  82.63±0.04  74.69±0.00  78.99±0.04  77.64±0.00  76.53±0.00 | *A. sinensis*  (Medicinal agarwood) |
| C012 | unknown | *Pt197FR*  *1535FR*  *5987FR*  *30515FR*  *125057FR*  *48959FR*  *55861FR*  *118649FR*  *148002FR*  *M856F21R* | T  A  G  A  A  A  G  A  A  G | 76.65±0.00  81.97±0.00  75.30±0.00  78.56±0.00  76.83±0.04  75.48±0.04  80.87±0.04  75.32±0.00  74.13±0.04  76.72±0.00 | Non-medicinal agarwood |
| C013 | unknown | *Pt197FR*  *1535FR*  *5987FR*  *30515FR*  *125057FR*  *48959FR*  *55861FR*  *118649FR*  *148002FR*  *M856F21R* | T  A  G  A  A  A  G  A  A  G | 76.56±0.04  82.08±0.04  75.44±0.00  78.49±0.00  76.94±0.04  75.64±0.04  80.81±0.04  75.46±0.00  74.28±0.00  76.70±0.04 | Non-medicinal agarwood |
| C014 | unknown | *48959FR*  *55861FR*  *118649FR*  *148002FR*  *M856F21R* | G  T  C  C  T | 76.16±0.04  80.31±0.00  75.85±0.29  75.11±0.04  76.48±0.04 | *A. agallocha*  (Medicinal agarwood) |
| C015 | unknown | *Pt197FR*  *1535FR*  *5987FR*  *30515FR*  *125057FR*  *48959FR*  *55861FR*  *118649FR*  *148002FR*  *M856F21R* | T  A  G  A  C/A  A  G  A  A  G | 76.94±0.05  82.21±0.04  75.57±0.00  78.73±0.10  77.07±0.04  75.64±0.08  80.72±0.04  75.59±0.00  74.41±0.00  76.66±0.11 | Non-medicinal agarwood |
| C016 | unknown | *Pt197FR*  *1535FR*  *5987FR*  *30515FR*  *125057FR*  *M856F21R* | G  C  T  G  C  T | 77.01±0.04  82.63±0.04  74.67±0.04  78.95±0.00  77.62±0.04  76.53±0.00 | *A. sinensis*  (Medicinal agarwood) |
| C017 | unknown | *Pt197FR*  *1535FR*  *5987FR*  *30515FR*  *125057FR*  *48959FR*  *55861FR*  *118649FR*  *148002FR*  *M856F21R* | T  A  G  A  A  A  G  A  A  G | 76.62±0.04  82.01±0.04  75.58±0.04  78.53±0.04  76.83±0.04  75.46±0.00  80.81±0.04  75.35±0.04  74.13±0.04  76.75±0.04 | Non-medicinal agarwood |
| C018 | unknown | *Pt197FR*  *1535FR*  *5987FR*  *30515FR*  *125057FR*  *48959FR*  *55861FR*  *118649FR*  *148002FR*  *M856F21R* | T  C  G  G/A  C/A  A  G  A  A  T/G | 76.71±0.00  82.36±0.00  75.56±0.14  78.51±0.08  77.34±0.08  75.42±0.04  80.50±0.04  75.32±0.00  74.13±0.04  76.66±0.00 | Mixed of non-medicinal agarwood |
| C019 | unknown | *Pt197FR*  *1535FR*  *5987FR*  *30515FR*  *125057FR*  *48959FR*  *55861FR*  *118649FR*  *148002FR*  *M856F21R* | T  G/A  Heterozygous  A  A  A  T  T/A  A  G | 76.65±0.00  82.25±0.04  75.58±0.04  78.76±0.04  77.36±0.04  75.59±0.00  78.78±0.04  75.37±0.04  74.13±0.16  76.99±0.07 | Non-medicinal agarwood |
| C020 | unknown | *Pt197FR*  *1535FR*  *5987FR*  *30515FR*  *125057FR*  *M856F21R* | G  C  T  G  C  T | 77.06±0.04  82.66±0.04  74.71±0.04  79.01±0.00  77.31±0.07  76.53±0.00 | *A. sinensis*  (Medicinal agarwood) |
