# Supplementary figures and images for: Successful development of molecular diagnostic technology combining mini-barcoding and high-resolution melting for traditional Chinese medicine agarwood species based on single-nucleotide polymorphism in the chloroplast genome
Source: Front Plant Sci. 2024 Jul 31;15:1405168. doi: 10.3389/fpls.2024.1405168 (PMC11322813; doi:10.3389/fpls.2024.1405168)

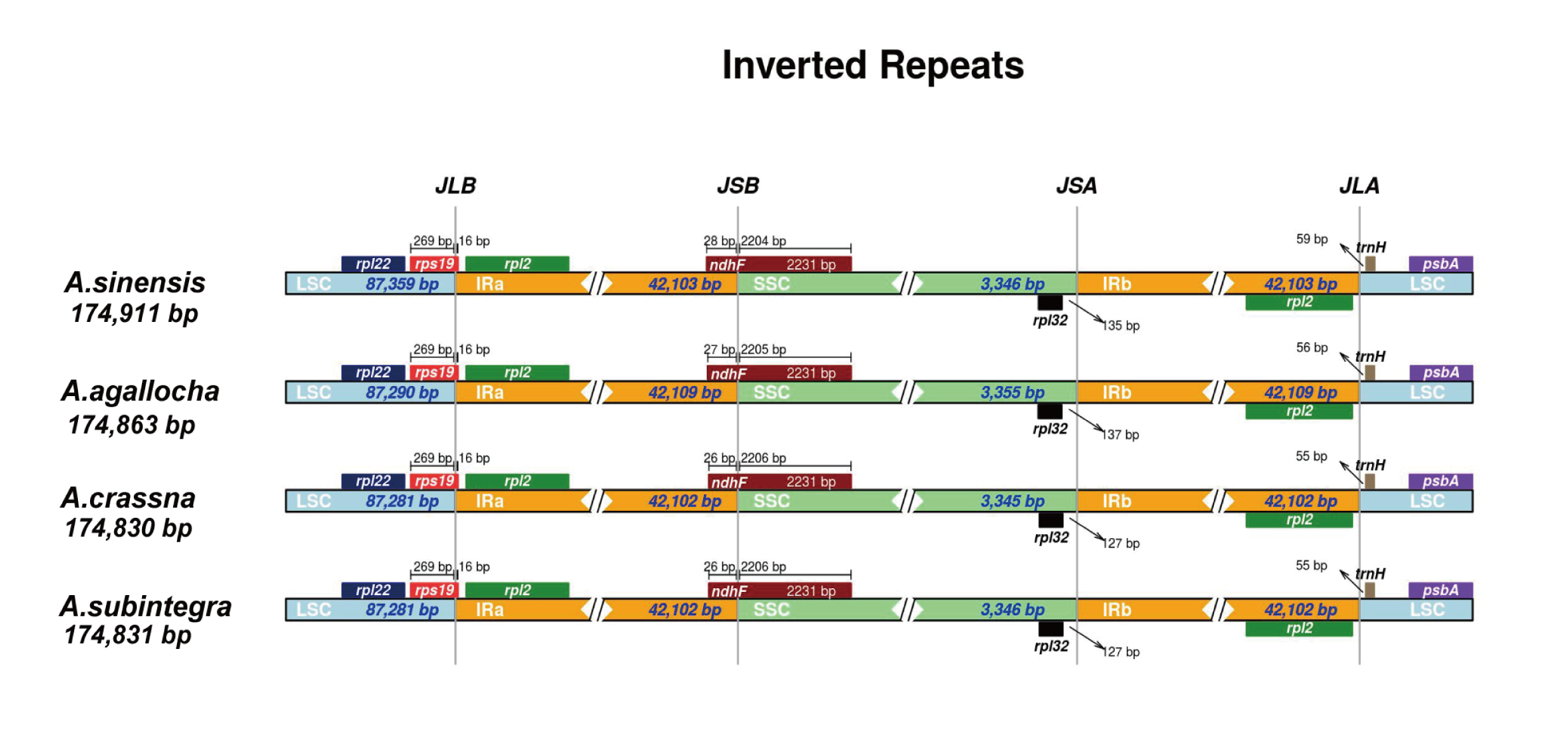

Supplement: Supplementary file 1 [file DataSheet_1.zip › Figure S1.TIF]

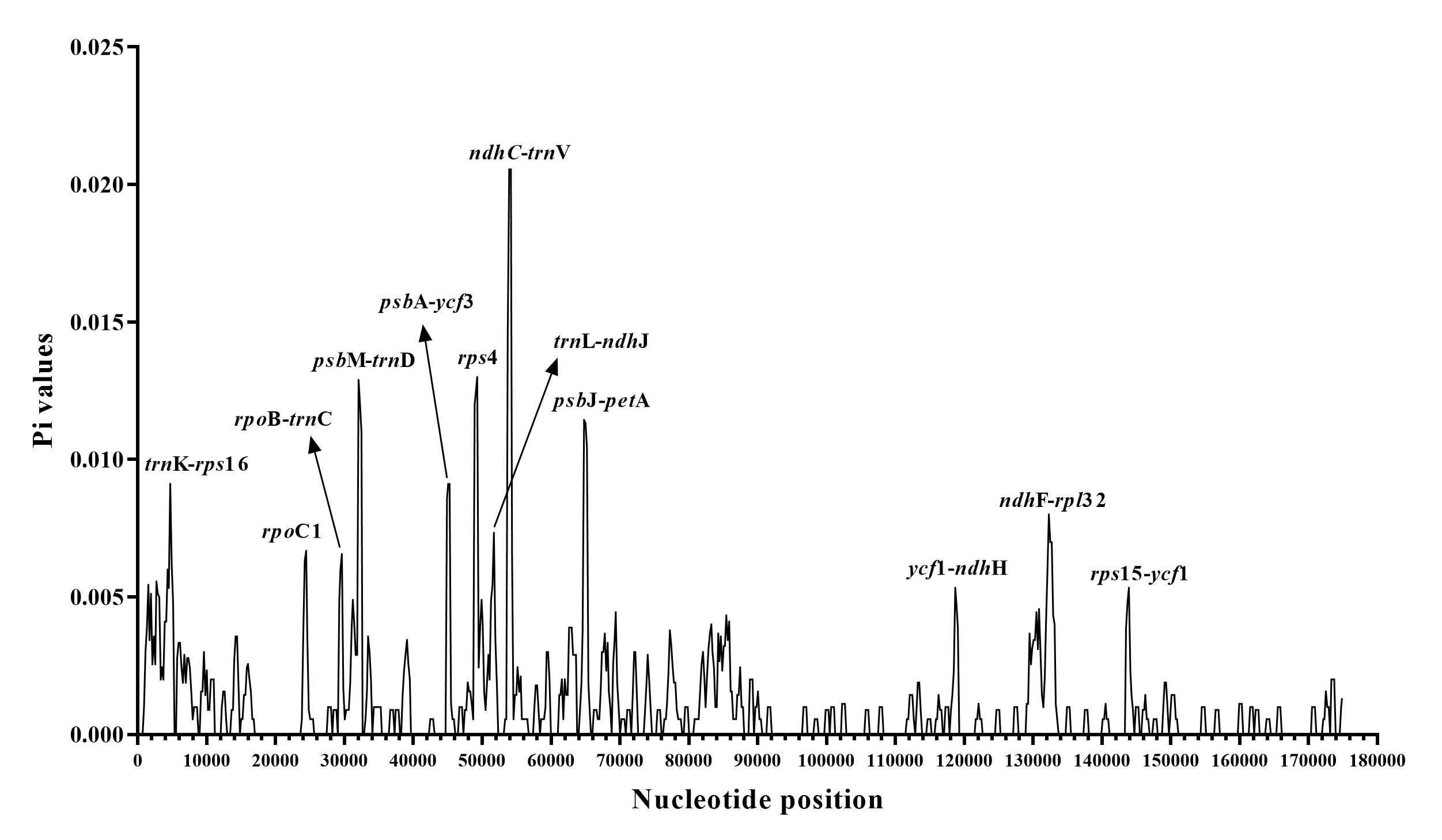

Supplement: Supplementary file 1 [file DataSheet_1.zip › Figure S2.TIF]
